# Prevalence and Risk Factors of Retinopathy in Type 1 Diabetes: A Cross-Sectional Study

**DOI:** 10.7759/cureus.47993

**Published:** 2023-10-30

**Authors:** Soumiya Berrabeh, Ouafae Elmehraoui, Siham Benouda, Imane Assarrar, Siham Rouf, Hanane Latrech

**Affiliations:** 1 Department of Endocrinology-Diabetology and Nutrition, Mohammed VI University Hospital Center, Oujda, MAR; 2 Department of Endocrinology-Diabetology and Nutrition, Faculty of Medicine and Pharmacy, Mohamed First University, Oujda, MAR; 3 Laboratory of Epidemiology, Clinical Research and Public Health, Faculty of Medicine and Pharmacy, Mohammed First University, Oujda, MAR

**Keywords:** microvascular complications, prevalence, risk factors, type 1 diabetes mellitus, diabetic retinopathy

## Abstract

Introduction

Diabetic retinopathy (DR) is a severe complication of diabetes. It remains a major cause of visual impairment and blindness, especially in young people. It is a silent affection that only becomes symptomatic at the onset of complications. Our study aimed to estimate the prevalence of retinopathy in patients with type 1 diabetes mellitus (T1DM) and evaluate the associated risk factors in our population.

Materials and methods

A descriptive and analytical study, with a cross-sectional study involving 359 patients with type 1 diabetes, was followed up in the Department of Endocrinology, Diabetology, and Nutrition of the University Hospital Center Mohammed VI Oujda, Morocco. Data were collected from medical records and analyzed by binary logistic regression using IBM Corp. Released 2012. IBM SPSS Statistics for Windows, Version 21.0. Armonk, NY: IBM Corp.

Results

The average age of our patients was 24.2 ± 11.4 years. The mean duration of diabetes was 11.8±4.4 years. The average glycated hemoglobin (HbA1c) at admission was 10.1 ± 2.4%. DR was found in 30% of patients, including 28.6% with minimal non-proliferative diabetic retinopathy (NPDR), 19.1% with moderate NPDR, 19.1% with severe NPDR, and 33.3% with proliferative DR. Patients with diabetic retinopathy appear to have a longer duration of diabetes (13.05±9.05 vs. 10.6±8.07 years). The longer duration of diabetes, neuropathy, and nephropathy was significantly associated with diabetic retinopathy (p=0.02, p=0.002, and p=0.0001, respectively).

Conclusion

The frequency of diabetic retinopathy increases with age, poor glycemic control, and the duration of diabetes. Therefore, cooperation between diabetologists and ophthalmologists is essential for making an early diagnosis and providing early treatment.

## Introduction

Diabetic retinopathy (DR) is one of the most frequent microvascular complications of diabetes and one of its most feared consequences; its incidence varies between 39% and 51% in different studies [1,2]. Patients with diabetes are at higher risk of visual impairment than non-diabetic ones [3]. Yet, DR continues to be one of the leading causes of preventable blindness in developed countries [4]. This visual impairment is due to the presence of proliferative diabetic retinopathy (PDR) and clinically significant macular edema (CSME) [5].

According to data from large worldwide epidemiologic studies, the incidence of type 1 diabetes mellitus (T1DM) has been increasing by 2% to 5% globally [6]. In parallel, this incidence increases the number of people at risk of developing DR, which increases with the duration of diabetes.

In the 1930s, many experts speculated that the only phenotype that might develop DR was middle-aged overweight adults with type 2 diabetes. After the discovery of insulin in 1921, doctors and researchers discovered that younger patients with T1DM began to live longer and that they were also developing retinopathy [7].

Despite the tremendous advances in medicine, medical technology, and devices, we cannot definitively prevent the visual impairment of DR because its diagnosis and management are often delayed. This delay is linked to the absence of visual symptoms during the early stages of the disease, hence the importance of screening [8]. The guidelines for screening for DR have been established by national professional organizations such as the American Diabetes Association (ADA) [9]. If vision-threatening retinopathy is diagnosed early, it offers the possibility of early treatment to save sight and maintain quality of life for people with diabetes.

Our study aims to estimate the prevalence of retinopathy in patients with T1DM and evaluate the associated risk factors in our population. This study has been reported following the STROBE criteria.

## Materials and methods

Study design

We carried out a descriptive and analytical study with a cross-sectional study involving 359 patients with T1DM, followed up in the Department of Endocrinology, Diabetology, and Nutrition. The ethical review committee at the Faculty of Medicine and Pharmacy approved the study design and protocol under the number 16/2020, and all involved patients provided written informed consent.

Study population

We included all patients with T1DM who were admitted to our department, with onset or unbalanced diabetes, young and adult patients, and with complete medical records. We excluded pregnant patients with T1DM, patients with type 2 diabetes, patients with incomplete medical records, and those who didn’t give their consent.

Study protocol

The mean age of patients was defined as the mean age at diagnosis of DR. The delay in the onset of signs of DR is the time from the diagnosis of diabetes to the onset of retinopathy.

Interview and physical examination data were collected from the patient’s medical records, including height, weight, body mass index (BMI), and blood pressure measurements. High blood pressure was defined as a blood pressure that is greater than or equal to 140/90 mmHg or lower in patients on antihypertensive treatment. The screening for diabetic nephropathy is determined by calculating the microalbumin/urine creatinine ratio and by measuring the glomerular filtration rate (GFR). Data regarding the presence or absence of diabetic neuropathy and macroangiopathic complications was also collected.

Glycemic control was assessed using data from the daily blood glucose self-monitoring and glycated hemoglobin (HbA1c) assays. The average HbA1c level for each patient was calculated from the HbA1c levels available in the medical records. Treatment regimens for insulin therapy were also noted.

DR screening and staging were performed during the regular ophthalmological assessments using a biomicroscopic examination of the fundus after pupillary dilation, supplemented by fundus photographs. Optical Coherence Tomography (OCT) was used for patients if the visual acuity (VA) was not corrected or if there were signs of DR or maculopathy on the fundus photographs for detecting macular oedema. Retinal OCT angiography was used at the stage of severe pre-proliferative retinopathy and proliferative retinopathy to search for neovessels and refer for laser treatment.

International Classification of Diabetic Retinopathy (2003), which classifies retinopathy into minimal, moderate, and severe non-proliferative retinopathy (NPDR), and proliferative diabetic retinopathy (PDR). Macular edema can be associated with all stages of DR. 

Statistical analysis

Statistical analysis was performed by IBM Corp. Released 2012. IBM SPSS Statistics for Windows, Version 21.0. Armonk, NY: IBM Corp. Descriptive statistics were mentioned for all variables: means, percentages, and standard deviation. Predisposing risk factors were analyzed by binary logistic regression. A value of p less than 0.05 was considered statistically significant.

## Results

**Figure 1:**
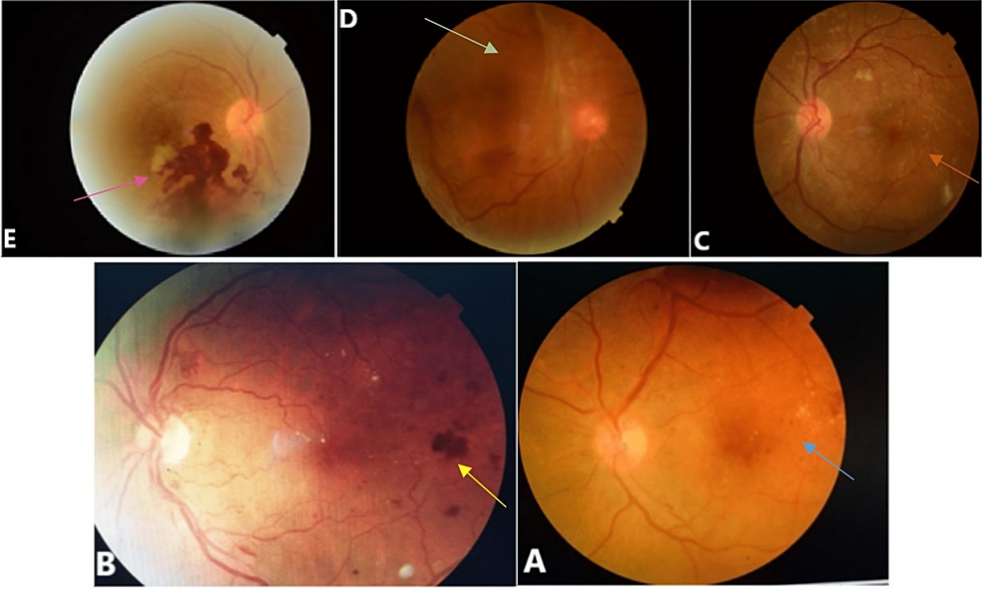
Fundoscopic images showing A. Minimal non-proliferative diabetic retinopathy with maculopathy (blue arrow), B. Proliferative diabetic retinopathy with papillary pallor (yellow arrow), C. Severe non-proliferative diabetic retinopathy on the left side (orange arrow), D. Proliferative diabetic retinopathy complicated by retinal detachment in the right eye (green arrow), E. Intraretinal hemorrhage on the right side (proliferative diabetic retinopathy) (pink arrow)

A total of 359 type 1 diabetic patients were included in this study. Thirty percent of patients had diabetic retinopathy, of which 28.4% had minimal non-proliferative retinopathy, 19.4% had moderate non-proliferative retinopathy, 19.4% had severe non-proliferative retinopathy, 32.8% had proliferative retinopathy, and 15.8% of patients had associated maculopathy (Figure 1).

The clinical characteristics of these patients with DR are detailed in Table 1. The mean age was 28.4 ± 13.4 years. There were no gender differences (50.8% were men). The mean duration of diabetes was more than five years in 46.5% of cases. The mean HbA1c was 10.5 ± 2.6%. Ninety-two percent of diabetic patients were on the basal-bolus insulin regimen. Subjects with DR were significantly older than those without and tended to have a higher BMI (Table 1).

**Table 1:** The clinical and demographic characteristics of patients DR: Diabetic retinopathy, A1c: Glycated hemoglobin

	Patients with DR	Patients without DR
Mean age (years)	28.4±13.4	20.1±9.4
Gender (male) (%)	50.8	51.5
Mean HbA1c (%)	10.5±2.6	9.8± 2.2
BMI (Kg/m2)	21.5±4.6	20.2±4.2
The mean duration of diabetes (years)	13.05± 9.05	10.6±8.07
Therapeutic regimen (basal-bolus regimen) (%)	92.1	83.8

The prevalence of DR increased dramatically with age (Tables 2, 3) and the longer duration of diabetes, in which 6.2% of patients had a duration less than five years and 79.4% had a duration longer than five years.

**Table 2:** The distribution of diabetic retinopathy and its stage according to age DR: Diabetic retinopathy, NPDR: Non-proliferative diabetic retinopathy, PDR: Proliferative diabetic retinopathy

Age (years)	Patients (%)	Stage of DR (%)
<15	11.9	minimal NPDR: 62.5
		moderate NPDR: 25
		severe NPDR: 12.5
15-18	13.5	minimal NPDR: 33.3
		moderate NPDR: 33.3
		severe NPDR: 22.2
		PDR: 11.2
>18	74.6	minimal NPDR: 22
		moderate NPDR: 16
		severe NPDR: 20
		PDR: 42

**Table 3:** The prevalence of different stages of diabetic retinopathy according to age DR: Diabetic retinopathy, NPDR: Non-proliferative diabetic retinopathy, PDR: Proliferative diabetic retinopathy

DR Stage	Age (years)	Percentage of DR (%)
Minimal NPDR	< 15	26.3
	15-18	15.8
	> 18	57.9
Moderate NPDR	< 15	15.4
	15-18	23.1
	> 18	61.5
Severe NPDR	< 15	7.7
	15-18	15.4
	> 18	76.9
PDR	< 15	0
	15-18	4.5
	>18	95.5

We used bivariate logistic regression to study the independent factors that influence the occurrence of diabetic retinopathy. We studied the following factors: age, sex, BMI, HbA1c, duration of diabetes, and association with nephropathy and neuropathy. Younger age was a protective factor for DR (aOR= 0.31, CI 95%: 0.13-0.72, p= 0.006). The duration of diabetes, neuropathy, and nephropathy were found to be independent risk factors for DR. (p: 0.02, p=0.002, p=0.000) (Table 4).

**Table 4:** Bivariate logistic regression model analyzing the risk factors for diabetic retinopathy aOR: Adjusted odds ratio, CI: Confidence interval. Statistically significant p<0.05.

Risk Variable	aOR	CI 95%	p-value
Age (<18 years / >18 years)	0.31	0.13-0.72	0.006
Sex (Male/Female)	0.68	0.33-1.37	0.28
BMI (<18.5kg/m2/<18.5kg/m2)	0.38	0.12-1.21	0.10
HbA1c	1.00	0.94-1.06	0.89
Vitamin D	1.55	0.62- 3.91	0.34
Duration of diabetes (<5years/ >5years)	1.00	1.001-1.009	0.02
Nephropathy (Yes/No)	11.7	3.77-36.31	0.000
Neuropathy (Yes/No)	14.21	2.67-75.48	0.002

Additionally, 38.8% of the cases had nephropathy, of which 16.6% were on dialysis, while 20.9% had associated neuropathy. Twenty-four percent of patients were hypertensive. For macroangiopathic complications, 3.2% had a history of stroke, 11.1% had lower limb arteriopathy, and 4.7% had ischemic heart disease.

Concerning treatment, 34% of patients had benefited from laser photocoagulation, 14.9% received intravitreous injections, and 8.6% had benefited from surgery.

## Discussion

An estimated 2.2 billion people in the world suffer from visual impairment or blindness, according to the global vision report published by the World Health Organization. Although these numbers are scary, 80% of the causes are treatable and/or preventable [10]. Diabetic retinopathy is one of the leading but preventable causes of blindness between the ages of 20 and 64 [11].

The increase in diabetes is associated with an increase in diabetic retinopathy; thus, it is important to identify the risk factors associated with diabetic retinopathy in youths with T1DM [12]. Risk factors evaluated in most studies include glycemic control (HbA1c), duration of diabetes, age, gender, and socio-economic status. Chew EY et al. found a significant correlation between increasing age and the risk of diabetic retinopathy (4.5%/year) in T1DM and a double risk in male patients. Unsurprisingly, elevated HbA1c was associated with a greater risk of diabetic retinopathy. In fact, every one-point increase in HbA1c increases the risk of developing diabetic retinopathy by 20%-30% in patients with T1DM [12].

In our series, the prevalence of DR was 6.2% in patients with a duration of diabetes mellitus (DM) less than five years and 79.4% in those with a duration greater than five years. Our results were similar to those reported by Z. Sehnaz Karadeniz [11], who noted that 5.4% of patients with a duration of diabetes less than five years developed DR, compared to 79.2% with a duration longer than 20 years. In the population-based Wisconsin Epidemiologic Study of Diabetic Retinopathy (WESDR) [13], the prevalence of DR among younger onset patients varied from 17% in patients with DM for less than five years to 97.5% for those with DM for 15 or more years. HbA1c as a measure of glycemic control and the duration of diabetes are the main modifiable risk factors for the development of DR reported in the literature [14].

Another common microvascular complication of diabetes is diabetic nephropathy. Experimental research shows a high association between pathological alterations in the retina and the renal vasculature. In the Renin-Angiotensin System study of the young diabetic type 1, the severity of retinopathy was positively correlated with biopsy-proven renal anatomic characteristics [15]. In addition, other epidemiological studies have consistently proved that DR is associated with microalbuminuria and clinical nephropathy independently of hypertension and other risk factors [15] (Table 5).

**Table 5:** The risk factors for diabetic retinopathy in the literature N: Number of patients, DR: Diabetic retinopathy, A1c: Glycated hemoglobin

	Patients (n)	Prevalence of DR (%)	Risk factors studied	P-value
Z. Sehnaz Karadeniz [11]	370	33.2	Duration of diabetes	p=0.00
S.M. Ng et al. [14]	237	11	Gender	p= 0.025
			Duration of diabetes	p= 0.01
			Age at diagnosis	p= 0.007
			Age at screening	p= 0.04
			Mean A1c	p= 0.004
C. Madeira [16]	233	43.3	Gender	p= 0.15
			Duration of diabetes	p< 0.001
			Age	p= 0.76
			Mean A1c	p< 0.001
Sophia Y. Wang et al. [17]	2240	20.1	Mean A1c	p<0.0001
			Age	p<0.001
			Male sex	p=0.19
			Race	p=0.6
Our study	359	30	Age	0.006
			Gender	0.28
			BMI	0.10
			HbA1c	0.89
			Vitamine D	0.34
			Duration of diabetes	0.02
			Nephropathy	0.000
			Neuropathy	0.002

Concerning the evaluation of predisposing factors for DR, in our study, patients with DR displayed a higher mean HbA1c than patients without DR. These findings are in concordance with previous studies [14,16,17]. Our results emphasize the need for DR screening during the first year following the diagnosis of type 1 diabetes in patients with very poor metabolic control.

On the other hand, we noted that patients whose age is less than 18 years had a lower risk of having diabetic retinopathy, which is underlined in some studies that have labeled age as a risk factor [14,17].

DR was equally frequent among males and females (p=0.5). No associations were found between the prevalence of DR and the age at T1DM diagnosis (p=0.28). The presence and severity of DR significantly increased with the increase in the duration of DM (p=0.02).

Furthermore, epidemiological studies [15,18,19] have consistently shown a correlation between DR, microalbuminuria, and clinical nephropathy, which has been significantly proven in our study (p=0.000). Patients with type 1 diabetes who had more severe retinopathy at baseline had a higher risk of nephropathy at four years, according to the population-based Wisconsin Epidemiologic Study of Diabetic Retinopathy (WESDR) [13]. In addition, the presence of specific signs of DR, such as retinal hemorrhages, microaneurysms, and absorbent cotton spots, was associated with an increased risk of kidney failure in the Atherosclerosis Risk in Communities (ARIC) study [18,19]. Similarly, the development of severe proteinuria and progression to renal failure were independently linked to the presence of retinopathy in the Cardiovascular Health Study [18]. These findings support the clinical recommendation to monitor renal function in diabetic patients with evidence of retinopathy and confirm that retinopathy and nephropathy share similar microvascular pathogenesis related to abnormal glucose metabolisms and other processes (inflammation, endothelial dysfunction) [20].

As opposed to nephropathy, the correlation between retinopathy and diabetic neuropathy is less evident. A few studies have demonstrated that retinopathy and the risk of neuropathy may be connected in diabetics. A longitudinal study of 264 diabetic patients found an association between the severity of microvascular disease, including retinal disease, and the severity of diabetic polyneuropathy [21]. Recently, the Australian Diabetes Obesity and Lifestyle Study, a population-based study of Australian adults aged 25 and above, reported a substantial connection between retinopathy and neuropathy in individuals without clinical diabetes but with glucose metabolism disorders [22]. In the WESDR [13], patients with severe NPDR or PDR had a greater risk of incident lower leg amputation compared to those without or with minimal retinopathy at baseline; this complication is partly attributed to diabetic somatic neuropathy [23,24]. In our study, we confirmed that diabetic retinopathy increases the risk of neuropathy, i.e., there is a statistically significant relationship between retinal injury and neuropathy (p=0.002) (Table 5).

Moreover, various studies suggest a pathogenic relationship between retinopathy and systemic vascular disease. Also, various genes related to DR have been implicated in the pathogenesis of cardiovascular disease. Additional studies, which are currently underway (e.g., the ARIC study and the multi-ethnic atherosclerosis study), will determine whether there is a potential mechanism linking diabetic microvascular disease (retinopathy) to macrovascular disease (atherosclerosis) [25-27].

In addition, some studies discuss vitamin D as a predictive factor for the onset and severity of diabetic retinopathy since vitamin D is recognized as an inhibitor of angiogenesis. However, in our study, we have not found a significant correlation between the level of vitamin D and diabetic retinopathy (Table 4).

The limitations of the study were that we couldn’t study the association between puberty and DR in young patients, mainly because of the small number of patients who presented during the period of puberty. The strength of the study is that it gives an insight into the prevalence of DR and its particularities in patients with T1DM living in a developing country.

## Conclusions

To conclude, diabetic retinopathy is a serious public health problem that diminishes the quality of life of patients with diabetes. It is asymptomatic in the early stages but may progress to a condition that compromises vision. Disease duration and the timing of puberty are the main risk factors for DR in youth with T1DM. Contrary to previous beliefs, DR is rather common in young patients with T1DM. Systematic diabetic retinopathy screening aims to reduce the risk of vision impairment and blindness because early detection is the key to preserving sight and preventing irreversible retinal damage.
